# Proportion of Thick versus Thin Melanomas as a Benchmarking Tool

**DOI:** 10.3390/jcm10235545

**Published:** 2021-11-26

**Authors:** Calogero Pagliarello, Serena Magi, Laura Mazzoni, Ignazio Stanganelli

**Affiliations:** 1 UO Multizonale Dermatologia, Ospedale “Santa Chiara”, 38122 Trento, Italy; 2 Istituto Scientifico Romagnolo per lo Studio e la Cura dei Tumori (IRST) IRCCS, 47014 Meldola, Italy; serena.magi@irst.emr.it (S.M.); laura.mazzoni@irst.emr.it (L.M.); ignazio.stanganelli@irst.emr.it (I.S.); 3 Section of Dermatology, Department of Medicine and Surgery, University of Parma, 43125 Parma, Italy

**Keywords:** dermoscopy, diagnostic accuracy, interpretive performance, melanoma, number needed to excise, number needed to treat

## Abstract

Background: The ratio of benign moles excised for each malignant melanoma diagnosed (number-needed-to-excise (NNE)) is a metric used to express the efficiency of diagnostic accuracy of melanoma. The literature suggests a progressive effort to reduce the NNE, thus raising concerns about missing early melanoma because the NNE does not capture the most significant outcome for melanoma prognosis, which is linked to the Breslow thickness. A lower NNE could reduce health costs related to melanoma diagnosis only if doing so does not increase the proportion of thicker melanomas. Objectives: The diagnostic performance by two tertiary referral centres using the NNE and proportion of thick (Breslow thickness > 1 mm) versus thin (Breslow thickness ≤ 1 mm) excised melanoma (thick/thin ratio: TTR) was compared to determine if a lower NNE is associated with a greater proportion of thicker melanoma. Combining TTR with NNE allows a better estimate of the effectiveness in melanoma diagnosis, assessing both the overall cost for a given pool of excised melanomas and costs due to unnecessary nevi excision at a particular dermatology centre. Methods: Demographic data and Breslow thickness of excised melanoma were extracted from patient histologic records at two referral centres for melanoma (Parma Dermatology Unit and Ravenna and Meldola Skin Cancer Unit, Istituto Scientifico Romagnolo per lo Studio e la Cura dei Tumori. IRCCS (IRST)) on all skin tumours excised between 2002 and 2011 and diagnosed as melanoma or melanocytic nevus. NNE and TTR were calculated and compared among the considered variables. Logistic regression was used to assess the contribution of each variable in predicting a higher TTR. Results: Data from 16,738 excised lesions were analysed. The IRST Unit reported a mean NNE of 4.6, whereas the Parma Unit excised 10.6 nevi for each melanoma. No statistically significant differences existed in the mean (IRST Unit, 0.56 ± 0.89 mm; Parma Unit, 1.07 ± 2.2 mm) and median (range) Breslow thickness (IRST Unit, 0.4 (9) mm; Parma Unit 0.4 (30) mm). The TTR between centres was significantly different (Parma Unit, 24%; IRST Unit, 12%; *p* < 0.001). Based on logistic regression, the diagnosing centre was the most powerful factor in determining a thickness of >1 mm among diagnosed melanomas (OR = 1.8; 95% CI, 1.2–2.7; *p* < 0.01), with all other factors being equal. The NNE decreased at both centres from younger-to-older patients, whereas the TTR increased simultaneously; however, the increase in TTR was non-significantly related to NNE reduction after adjusting for confounders (age, gender, and localization). Conclusions: A better diagnostic performance is capable of reducing the NNE and TTR, i.e., unnecessary excisions of melanocytic nevi can be reduced without increasing the risk of overlooking melanomas. The TTR, in addition to the NNE, allows stakeholders to better estimate the effectiveness in melanoma diagnosis because both overall costs for a given pool of excised melanomas and costs due for unnecessary nevi excision at a particular dermatology centre can be compared.

## 1. Introduction

The ratio of benign moles excised for each malignant melanoma diagnosed, i.e., the number-needed-to-excise (NNE), is regarded as a useful indicator of diagnostic accuracy [1,2,3,4,5]. NNE allows comparison between general practitioners, plastic surgeons, dermatologists, and skin cancer clinics for accuracy of melanoma diagnosis, suggesting a better diagnostic performance by dermatologists than other specialists. The literature suggests a progressive effort to reduce the NNE. It has been reported that dermoscopy [6,7,8,9,10], and more recently reflectance confocal microscopy (RCM) [11,12], have the potential to further reduce the NNE. Nevertheless, the NNE does not capture the most significant outcome for melanoma prognosis, which is linked to the Breslow thickness. Moreover, with respect to efficiency, i.e., the cost of healthcare to the outputs or benefits obtained, patients with intermediate thickness melanomas (those with a Breslow thickness > 1 mm) routinely undergo sentinel lymph node biopsy (SLNB) and a more intensive follow-up protocol, with a significant increase in overall cost in addition to possible increased morbidity and mortality. Therefore, a lower NNE could reduce health costs related to melanoma diagnosis only if doing so does not increase the proportion of thicker melanomas. The proportion of thick (Breslow thickness > 1 mm) versus thin (Breslow thickness ≤ 1 mm) excised melanomas (thick/thin ratio: TTR) allows stakeholders to better estimate the effectiveness in melanoma diagnosis, i.e., the benefits of healthcare measured by improvements in health because both overall costs for a given pool of excised melanomas and costs due for unnecessary nevi excision at a particular dermatology centre could be compared. Surprisingly, in the above-cited papers, the TTR could be deduced only in the study by Argenziano et al. [7], with a 28% TTR in a specialized clinical setting compared to a TTR of 14% among non-specialized clinical settings (Table 1 (page 56)). Thus, the need for larger margins of diagnostic safety when managing melanoma raises concerns of an unjustifiable NNE reduction if assessed without considering Breslow thickness. Furthermore, it is known that many patient-related factors influence the NNE, with higher values among females, younger patients and lesions localized on the trunk. Nevertheless, little is known about factors affecting the TTR and the possible relationship to the NNE.

To provide insight into this issue, we performed a retrospective analysis of data on pigmented lesions excised over a 10-year period (2002–2011) at two referral centres (Parma Dermatology Unit of Parma Medical School and Ravenna and Meldola Skin Cancer Unit, Istituto Scientifico Romagnolo per lo Studio e la Cura dei Tumori IRCCS (IRST)) to compare the TTRs and NNEs and analyse the possible reciprocal interaction and relationship with patient characteristics and lesion localization.

## 2. Material and Methods

Demographic data, such as patient gender, age, lesion location (head and neck, trunk, arms, legs, acral), and for melanoma cases the Breslow thickness, were extracted from patient histologic records at two large referral centres for melanoma (Parma Dermatology Unit and IRST) on all skin tumours excised between 2002 and 2011 that were diagnosed as melanoma or melanocytic nevus. These centres serve as tertiary referral centres for melanoma diagnosis for an inner-city population of approximately 160,000. The referrals are usually from primary and secondary medical care. At both centres, dermoscopy and digital dermoscopy follow-up are currently used. Furthermore, at the IRST, reflectance confocal microscopy became available in 2009. The TTR and the NNE were calculated and compared among the considered variables. Logistic regression analysis was used to assess each variable contribution in predicting a higher TTR. Data were analysed with IBM SPSS Statistics 21 (IBM SPSS Statistics for Windows, version 21.0 (released in 2012); IBM Corp., Armonk, NY, USA). A *p* value < 0.05 was considered statistically significant.

## 3. Results

A total of 16,738 excised lesions were analysed. Values for the variables of interest are shown in Table 1. Both nevi and melanoma excised from the two centres differed for patient age and location, whereas data were comparable for gender distribution.

The IRST reported a mean NNE of 4.6, whereas the Parma Dermatology Unit excised 10.6 nevi for each melanoma. The NNE was higher in both centres among females, and considering localization, the highest NNT was observed for trunk lesions. The TTR resulted in statistically significant differences between the two centres (IRST TTR, 12% vs. Parma TTR, 24%; *p* < 0.001). Considering each location, a significant difference in TTR was found for lesions excised from arms (*p* < 0.01). Notably, no statistically significant differences existed in the mean (IRST, 0.56 ± 0.89 mm; Parma, 1.07 ± 2.2 mm) and median (range) Breslow thickness (IRST, 0.4 (9) mm; Parma, 0.4 (30) mm). A higher proportion of thick melanomas existed among male patients in Parma (*p* < 0.001), whereas no differences were observed at the IRST. Based on multivariate analysis, the diagnosing centre was the most powerful factor in determining a thickness of > 1 mm among diagnosed melanomas, all other factors being equal (Table 2). Based on logistic regression analysis, the Parma Centre (odds ratio (OR), 1.8; 95% confidence interval (CI), 1.2–2.7; *p* < 0.01), male gender (OR, 1.5; 95% CI, 1.2–2; *p* < 0.01), age (OR, 1.2; 95% CI, 1.1–1.3; *p* < 0.001 for each 10-year increase), and location using the head and neck as the reference (trunk (OR, 1.5; 95% CI, 0.9–2.2), arms (OR, 1.8; 95% CI, 1.1–2.9), legs (OR, 2; 95% CI, 1.2–3), and acral (OR, 5.6; 95% CI, 3.3–9.6); *p* < 0.001) were associated with a thick melanoma diagnosis.

**Figure 1:**
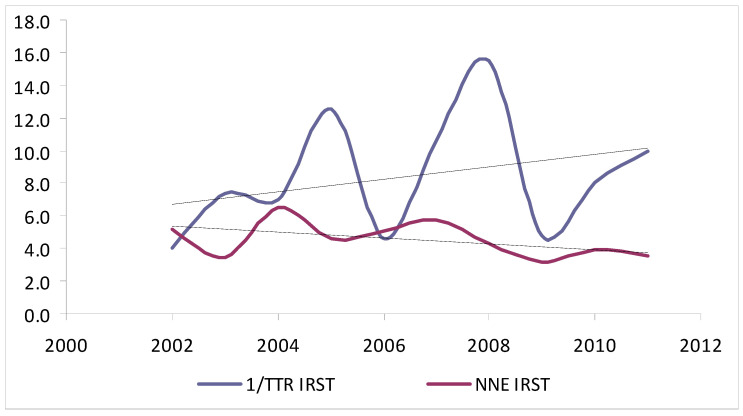
Trends for NNE and proportion of thin versus thick melanomas excised at IRST from 2002 to 2011.

**Figure 2:**
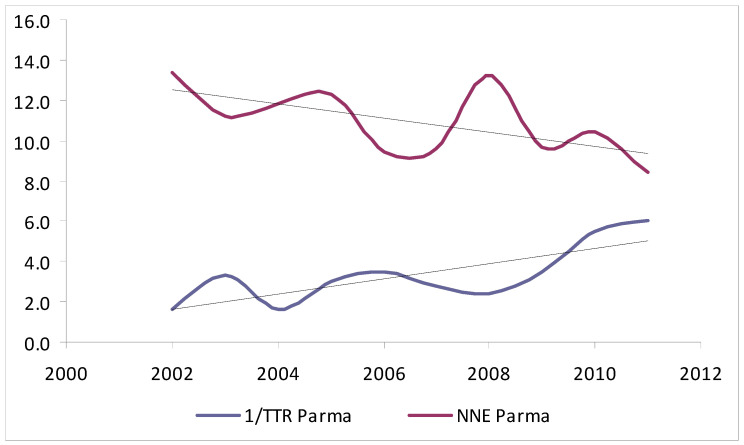
Trends for NNE and proportion of thin vs. thick melanomas excised at Parma from 2002 to 2011.

In analysing the NNT and TTR per 10-year age groups (Table 3), significant differences were observed among the two centres in excised nevi (*p* < 0.001) and overall melanomas (*p* < 0.05), but not for thin or thick melanomas. Interestingly, the NNE decreased in both centres from younger-to-older patients (IRST, from 82 among patients 10–19 years of age to 0.3 in patients 80–89 years of age; Parma, from 147 in patients 10–19 years of age to 0.55 in patients 90–99 years of age), whereas the TTR increased simultaneously, with the lowest value (Parma, 12.7%; IRST, 8%) among patients 20–29 years of age and the highest value among patients 80–89 years of age (Parma, 50%; IRST, 31%). Of note, the increase in TTR resulted in a non-significant reduction in NNE after adjusting for confounders (age, gender, and location). Finally, considering the year of excision (Table 4), the NNT decreased from 2002 to 2011, with a concurrent increase in the proportion of thin melanomas at both centres, but this trend was only statistically significant at Parma (*p* < 0.01; Figure 1 and Figure 2).

## 4. Discussion

The healthcare costs associated with melanoma screening have been estimated through the NNE based on the assumption that higher unnecessary excision of benign lesions would also increase medical costs. Identification of the costs and the key resource utilization drivers will assist health system administrators in making informed policy decisions. Nevertheless, assessing performance regardless of outcome could be deceptive. The greater burden of healthcare costs related to melanomas is linked to those cases which are diagnosed at advanced stages. Accordingly, our efforts should be aimed towards the early detection of melanomas. Aside from increasing overall morbidity and mortality, a delayed melanoma diagnosis is likely to increase melanoma-related costs because of higher resource utilization (increased hospitalizations, cancer clinic visits, systemic therapy costs, and home care) and income loss. By sub-stage, the 10-year survival ranged from 93% for stage IA (Breslow thickness ≤ 1 mm without ulceration and < 1 mitosis per mm^2^) to 39% for stage IIC melanoma (Breslow thickness > 4 mm and ulcerated) [13]. Moreover, the American Joint Committee on Cancer Melanoma Staging Committee recommends that sentinel lymph node biopsy be performed as a staging procedure in patients with Breslow thicknesses > 1 mm and clinically uninvolved regional lymph nodes because this information is considered useful in planning subsequent treatments and follow-up regimens [13]. SLNBs have an estimated total cost between EUR 9486.57 and EUR 10,471.29 [14], which exceeds >50 outpatient surgical excisions. Moreover, follow-up guidelines are based on stage and tumour thickness. According to the Swiss and German guidelines [15,16], no lymph node sonography is recommended for stage I (Breslow < 1.0 mm). In contrast, abdominal sonography, chest x-ray, whole-body imaging by computed tomography, and/or positron emission tomography scans every 3–12 months, and annual magnetic resonance imaging scans of the brain are recommended in the first 5 years of follow-up in patients with stage IIB-IV melanomas according to the National Comprehensive Cancer Network [17]. For these reasons, NNE is of limited utility as a proxy for estimating melanoma diagnostic efficacy without also considering the Breslow thickness of excised melanomas for a specified number of benign moles removed. Indeed, the thickness of tumours removed before and after education programs is a short-term measure of outcome frequently published in the literature. An increase in the proportion of tumours removed in the very thin categories is often cited as evidence of a successful response [18].

It is conceivable that aiming for the diagnosis of early melanoma to reduce unnecessary excision of benign lesions involves distinct interpretative approaches, skills, and thresholds for noting abnormalities as occurs for interpreting mammography [19]. Thus, it seems reasonable that raising the cut-off value for nevus atypia requested to be excised possibly decreases the proportion of early melanomas detected, in which atypia is still comparable to benign nevi. Using only NNE to assess dermatologist performance will result in coarse, biased conclusions. Hypothetically, a centre could easily reach an NNE equal to the least possible value, i.e., an NNE = 1, by removing only obvious melanomas. An NNE raises concerns about missing early melanomas excised to evaluate the ability to detect early disease, and the ratio of invasive melanomas to melanoma in situ has been recently proposed as a marker of sensitivity measures [20,21]. Nevertheless, this ratio does not fully take into account melanoma-staging considerations and resources allocated for a given melanoma diagnosis, equating stage T1 melanoma of 0.3 mm Breslow depth, which requires further wide excision without the need for annual computerized tomography scans to the worse stage T4 melanoma, a 5 mm Breslow depth, which necessitates an SLNB and more intensive follow-up involving expensive imaging procedures.

We propose that the TTR is a useful ratio to be used in addition to the NNE as a more meaningful benchmark comparison between centres for melanoma diagnosis. The TTR allows a better estimation of melanoma screening costs because the TTR includes melanoma staging using the cut-off value calculated by the AJCC for recommending SNLB based on prognostic factors. In contrast, the TTR, in addition to the NNE, may facilitate the assessment of the net economic benefits of a specified diagnostic performance, thus allowing a direct comparison of costs required for a given pool of melanomas for SLNB versus a given number of unnecessary excisions, weighing data accordingly. It has been reported that the costs avoided by diagnosing melanoma just 6 months earlier justify 170 biopsies per melanoma identified [22]. Therefore, efforts to penalize “unnecessary” biopsies may be economically counterproductive without considering outcomes. The Breslow mean value of diagnosed melanomas could also have been considered as an outcome measure for diagnostic performance. Nevertheless, in our study, the TTR was more sensitive than the mean and median Breslow thickness values in identifying differences among the two centres with respect to outcome.

The current study reported NNE and TTR for two dermatology referral centres from 2002 to 2011, suggesting that a better diagnostic performance is capable of reducing both ratios, i.e., unnecessary excisions of melanocytic nevi can be reduced without increasing the risk of overlooking melanomas, thus delivering the effectiveness and efficiency in melanoma diagnosis. In accordance with previous reports, the NNE decreased with time. At the same time, a reduction in TTR was observed at both centres, thus confirming that a reduction in NNE was obtained without an increase in missed early melanomas, as supposed from previous reports [21]. Technical improvements in dermoscopic equipment, especially with respect to digital follow-up examinations, could partly justify improvement in diagnostic performance [23]. In fact, the IRST was a top performer according to both types of ratios, but when adjusted for other variables, RCM availability did not fully explain performance. The results suggest that a greater diagnostic effort should be addressed among male and older patients in which an increased proportion of melanoma resulted in a delayed diagnosis after adjusting for melanoma location.

A limit of our study was that both the NNE and TTR are not linked to particular diagnosing dermatologists. In this regard, IRST performance reflected the work of the senior author (IS), whereas the Parma results were the sum of the performance of more than one dermatologist (12 in all), thus increasing variability, as occurs among radiologists as the diagnostic work-up volume is linked to screening mammography performance [24]. Another limit was that both TTR and NNE are affected by the underlying incidence of melanoma, referral patterns from the community, the community awareness about melanoma, and patient populations being examined.

## 5. Conclusions

The incidence of malignant melanoma is on the rise, and an increased number of skin-related healthcare visits is expected; therefore, diagnostic performance in melanoma diagnosis is of increased importance. To reduce health care resources, pigmented lesion management should be addressed in a specialized setting, as occurs for breast cancer.

Setting minimum performance cut-off values for the NNE and TTR could allow dermatologists to be involved in internal benchmark activities for clinical practice improvement efforts.

## Figures and Tables

**Table 1:** The number of excised nevi and melanomas, and NNE and TTR ratios, according to gender and location at two referral centres (IRST and Parma) from 2002 to 2011.

	IRST	Parma	*p*
OVERALL EXCISED LESION	1604	15,134	
EXCISED NEVI	1308	13,828	
Age (mean ± SD)	38.57 ± 13.77	36.97 ± 15.6	<0.001
Gender (%)			
Male	553 (42%)	5633 (41%)	NS
Female	755 (58%)	8195 (59%)	
Location (%)			
Head and neck	110 (8.5%)	2080 (16.5%)	<0.001
Trunk	846 (65.5%)	7029 (55%)	
Arms	90 (7%)	1177 (9.5%)	
Legs	212 (16.5%)	1400 (11%)	
Acral	34 (2.5%)	1033 (8%)	
EXCISED MELANOMA	296	1306	
Gender (%)			
Male	139 (47%)	592 (45%)	NS
Female	157 (53%)	714 (55%)	
Mean age ± SD	53.8 ± 18	56.3 ± 18	<0.05
Mean Breslow ± SD	0.56 ± 0.89	1.07 ± 2.2	NS
Median Breslow (range)	0.4 (9)	0.4 (30)	NS
Melanoma type (%)			<0.001
In situ	94 (31.8%)	510 (39.1%)	
Breslow ≤ 1 mm	166 (56.1%)	482 (36.9%)	
Breslow >1 mm	36 (12.2%)	314 (24%)	
Thin melanoma (%)	260 (87.8%)	992 (76%)	<0.001
Thick melanoma (%)	36 (12.2%)	314 (24%)	
Gender (%)			
Male			
Thin melanoma	124 (89.2%)	421 (71.1%) *	
Thick melanoma	15 (10.8%)	171 (28.9%) *	
Female			
Thin melanoma	136 (86.6%)	571 (80%) *	
Thick melanoma	21 (13.4%)	143 (20%) *	
Location (%)			<0.001
Head and neck	29 (10%)	220 (18%)	
Trunk	156 (53%)	458 (38%)	
Arms	41 (14%)	171 (14%)	
Legs	64 (22%)	280 (23%)	
Acral	3 (1%)	90 (7%)	
Head and neck (%)			NS
Thin melanoma	26 (89.7%)	178 (80.9%)	
Thick melanoma	3 (10.3%)	42 (19.1%)	
Trunk (%)			NS
Thin melanoma	136 (87.2%)	368 (80.3%)	
Thick melanoma	20 (12.8%)	90 (19.7%)	
Arms (%)			<0.01
Thin melanoma	38 (92.7%)	125 (73.1%)	
Thick melanoma	3 (7.3%)	46 (26.9%)	
Legs (%)			NS
Thin melanoma	55 (85.9%)	218 (77.9%)	
Thick melanoma	9 (14.1%)	62 (22.1%)	
Acral (%)			NS
Thin melanoma	3 (100%)	43 (47.8%)	
Thick melanoma	0 (0%)	47 (52.2%)	
OVERALL NNE	4.42	10.6	
Male NNE	3.98	9.52	
Female NNE	4.81	11.48	
Head and neck NNE	3.79	9.45	
Trunk NNE	5.42	15.35	
Arms NNE	2.19	6.88	
Legs NNE	3.31	5	
Acral NNE	11.33	11.47	

Totals may vary because of missing values. Legend: SD: standard deviation; thin melanoma: Breslow ≤ 1 mm, including in situ melanoma; thick melanoma: Breslow > 1 mm; NNE: number-needed-to-excise; NS: statistically not significant; * Comparison between males and females for a given centre, *p* < 0.001.

**Table 2:** Univariate and multivariate analyses (N = 16,738) in predicting thick melanoma diagnosis.

	Thick vs. Thin			Thick vs. Thin		
	Unadjusted			Adjusted		
	OR	95% CI	*p*	OR	95% CI	*p*
Centre						
IRST	1	–	<0.001	1	–	<0.01
Parma	2.3	1.6–3.3		1.8	1.2–2.7	
Gender						
Female	1	–	<0.001	1	–	<0.01
Male	1.5	1.1–1.9		1.5	1.2–2	
Location						
Head and neck	1	–	<0.001	1	–	<0.001
Trunk	0.9	0.7–1.4		1.5	0.9–2.2	
Arms	1.4	0.9–2.1		1.8	1.1–2.9	
Legs	1.2	0.8–18		2	1.2–3	
Acral	4.6	2.7–7.8		5.6	3.3–9.6	
Age (for each ten-year increase)	1.2	1.2-1.3	<0.001	1.2	1.1–1.3	<0.001
RCM			<0.01			NS
No	3.4	1.3–8.5		1.9	0.7–5.2	
Yes	–	–		–	–	

Abbreviations: OR: odds ratio, CI: confidence interval, NS: statistically not significant, RCM: reflectance confocal microscopy.

**Table 3:** The number of excised nevi and melanomas, and NNE and TTR ratios, according to age group at two referral centres (IRST and Parma) from 2002 to 2011 (N = 16,738).

	0–9	10–19	20–29	30–39	40–49	50–59	60–69	70–79	80–89	90–99	*p*
EXCISED NEVI											
IRST (%)	14 (1.1)	82 (6.3)	213 (16.3)	438 (33.6)	319 (24.5)	125 (9.6)	72 (5.5)	34 (2.6)	7 (0.5)	0 (0)	<0.001
Parma (%)	218 (1.6)	1622 (11.8)	2679 (19.4)	3916 (28.4)	2647 (19.2)	1361 (9.9)	890 (6.5)	355 (2.6)	84 (0.6)	10 (0.1)	
EXCISED MELANOMA											
Overall											
IRST (%)	0 (0)	1 (0.3)	27 (9.2)	54 (18.3)	56 (19)	41 (13.9)	37 (12.5)	57 (19.3)	21 (7.1)	1 (0.3)	<0.05
Parma (%)	0 (0)	11 (0.8)	71 (5.4)	207 (15.8)	218 (16.7)	210 (16.1)	217 (16.6)	222 (17)	132 (10.1)	18 (1.4)	
Thin											
IRST (%)	0 (0)	1 (0.4)	25 (9.7)	46 (17.8)	51 (19.7)	36 (13.9)	34 (13.1)	49 (18.9)	16 (6.2)	1 (0.4)	NS
Parma (%)	0 (0)	8 (0.8)	63 (6.4)	182 (18.3)	169 (17)	160 (16.1)	166 (16.7)	152 (15.3)	88 (8.9)	4 (0.4)	
Thick											
IRST (%)	0 (0)	0 (0)	2 (5.6)	8 (22.2)	5 (13.9)	5 (13.9)	3 (8.3)	8 (22.2)	5 (13.9)	0 (0)	NS
Parma (%)	0 (0)	3 (1)	8 (2.5)	25 (8)	49 (15.6)	50 (15.9)	51 (16.2)	70 (22.3)	44 (14)	14 (4.5)	
NNE IRST	NA	82	7.9	8.1	5.7	3	1.9	0.6	0.3	NA	<0.001
Thick/thin (TTR) IRST	n/a	n/a	0.08	0.17	0.10	0.14	0.09	0.16	0.31	n/a	NS
NNE Parma	NA	147	37.7	18.9	12.1	6.5	4.1	0.6	0.6	0.55	<0.001
Thick/Thin (TTR) Parma	n/a	0.37	0.13	0.14	0.29	0.31	0.30	0.45	0.50	3.33	<0.001
Excised melanoma IRST											
Thin (%)	0 (0)	1 (0.4)	25 (9.7)	46 (17.8)	51 (19.7)	36 (13.9)	34 (13.1)	49 (18.9)	16 (6.2)	1 (0.4)	NS
Thick (%)	0 (0)	0 (0)	2 (5.6)	8 (22.2)	5 (13.9)	5 (13.9)	3 (8.3)	8 (22.2)	5 (13.9)	0 (0)	
Excised melanoma Parma											
Thin (%)	0	8 (0.8)	63 (6.4)	182 (18.3)	169 (17)	160 (16.1)	166 (16.7)	152 (15.3)	88 (8.9)	4 (0.4)	<0.001
Thick (%)	0	3 (1)	8 (2.5)	25 (8)	49 (15.6)	50 (15.9)	51 (16.2)	70 (22.3)	44 (14)	14 (4.5)	

Totals may vary because of missing values. Legend: Thin melanoma: Breslow ≤ 1 mm, including in situ melanoma; Thick melanoma: Breslow > 1 mm; NNE: number-needed-to-excise; NS: statistically not significant.

**Table 4:** The number of excised nevi and melanoma, and NNE and TTR ratios, according to excisions per year at two referral centres (IRST and Parma; N = 16,738).

	2002	2003	2004	2005	2006	2007	2008	2009	2010	2011	*p*
EXCISED NEVI											
IRST (%)	157 (12)	142 (10.9)	157 (12)	124 (9.5)	144 (11)	131 (10)	141 (10.8)	92 (7)	105 (8)	115 (8.8)	<0.001
Parma (%)	765 (5.5)	1018 (7.4)	1235 (8.9)	1637 (11.8)	1415 (10.2)	1482 (10.7)	1589 (11.5)	1537 (11.1)	1553 (11.2)	1597 (11.5)	
EXCISED MELANOMA											
Overall											
IRST (%)	30 (0)	42 (0.3)	24 (9.2)	27 (18.3)	28 (19)	23 (13.9)	33 (12.5)	29 (19.3)	27 (7.1)	33 (0.3)	<0.001
Parma (%)	57 (4.4)	91 (7)	105 (8)	133 (10.2)	150 (11.5)	154 (11.8)	120 (9.2)	158 (12.1)	149 (11.4)	189 (14.5)	
Thin											
IRST (%)	24 (9.2)	37 (14.2)	21 (8.1)	25 (9.6)	23 (8.8)	21 (8.1)	31 (11.9)	24 (9.2)	24 (9.2)	30 (11.5)	<0.001
Parma (%)	35 (3.5)	70 (7.1)	64 (6.5)	100 (10.1)	117 (11.8)	113 (11.4)	85 (8.6)	120 (12.1)	126 (12.7)	162 (16.3)	
Thick											
IRST (%)	6 (16.7)	5 (13.9)	3 (8.3)	2 (5.6)	5 (13.9)	2 (5.6)	2 (5.6)	5 (13.9)	3 (8.3)	3 (8.3)	NS
Parma (%)	22 (7)	21 (6.7)	41 (13.1)	33 (10.5)	33 (10.5)	41 (13.1)	35 (11.1)	38 (12.1)	23 (7.3)	27 (8.6)	
NNE IRST	5.2	3.4	6.5	4.6	5.1	5.7	4.3	3.2	3.9	3.5	NS
TTR IRST	0.25	0.14	0.14	0.08	0.22	0.09	0.06	0.21	0.12	0.10	NS
NNE Parma	13.4	11.2	11.8	12.3	9.4	9.6	13.2	9.7	10.4	8.4	<0.01
TTR Parma	0.62	0.30	0.62	0.33	0.29	0.36	0.42	0.31	0.18	0.17	<0.01
Excised melanoma IRST											
Thin	24 (9.2)	37 (14.2)	21 (8.1)	25 (9.6)	23 (8.8)	21 (8.1)	31 (11.9)	24 (9.2)	24 (9.2)	30 (11.5)	NS
Thick	6 (16.7)	5 (13.9)	3 (8.3)	2 (5.6)	5 (13.9)	2 (5.6)	2 (5.6)	5 (13.9)	3 (8.3)	3 (8.3)	
Excised melanoma Parma											
Thin	35 (3.5)	70 (7.1)	64 (6.5)	100 (10.1)	117 (11.8)	113 (11.4)	85 (8.6)	120 (12.1)	126 (12.7)	162 (16.3)	<0.001
Thick	22 (7)	21 (6.7)	41 (13.1)	33 (10.5)	33 (10.5)	41 (13.1)	35 (11.1)	38 (12.1)	23 (7.3)	27 (8.6)	

Totals may vary because of missing values. Legend: Thin melanoma: Breslow ≤ 1 mm, including in situ melanoma; Thick melanoma: Breslow > 1mm; NNE: the number needed to excise; NS: statistically not significant.

## Data Availability

The data presented in this study are available on request from the corresponding author. The data are not publicly available because they could compromise the privacy of research participants.
